# Supplementary figures and images for: Prognostic significance of SH2D5 expression in lung adenocarcinoma and its relation to immune cell infiltration
Source: PeerJ. 2023 May 9;11:e15238. doi: 10.7717/peerj.15238 (PMC10178299; doi:10.7717/peerj.15238)

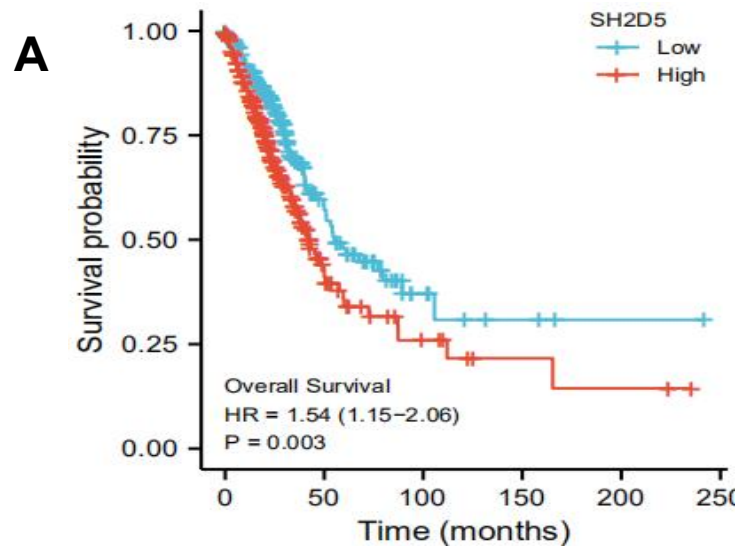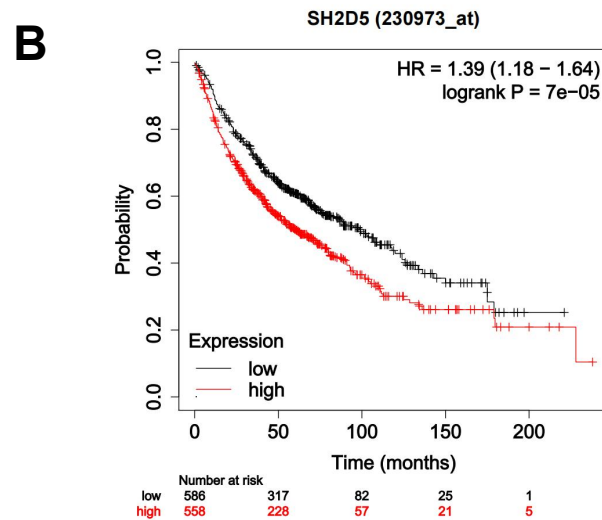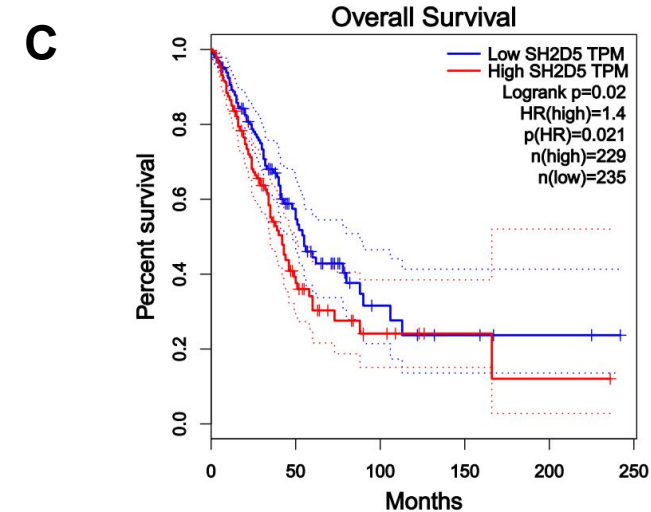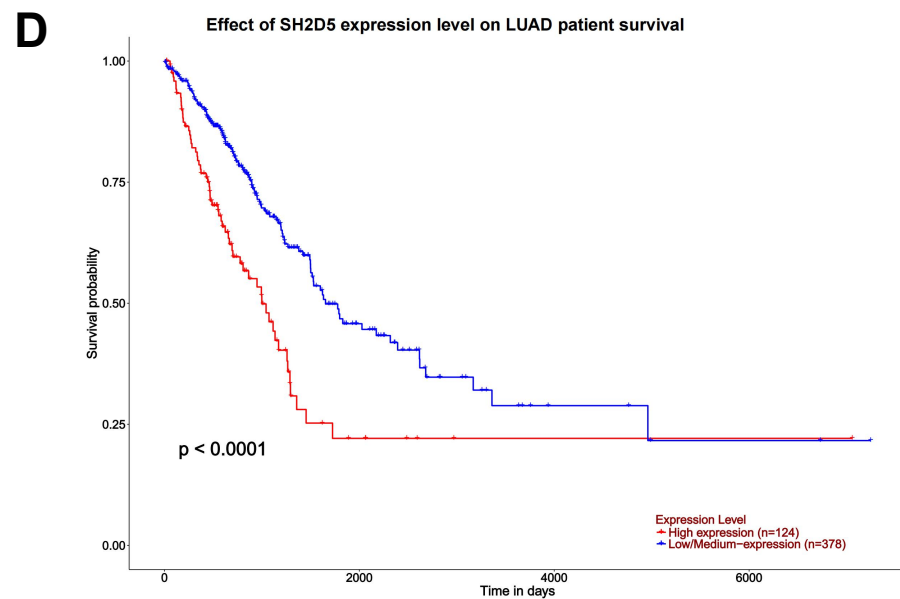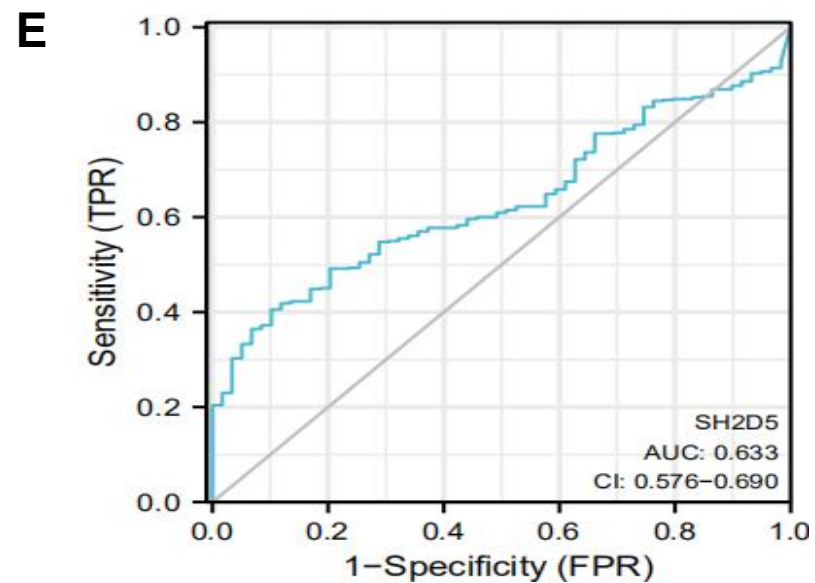

Supplement: Figure S1 — A.D. The correlation between SH2D5 and prognosis was verified by KM-plot, GEPIA, and UALCAN databases. F. ROC curves verified diagnostic efficacy of SH2D5 for LUAD by R. [file peerj-11-15238-s003.pdf]

**A**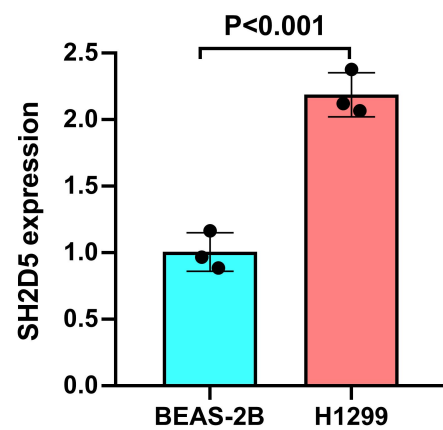**B**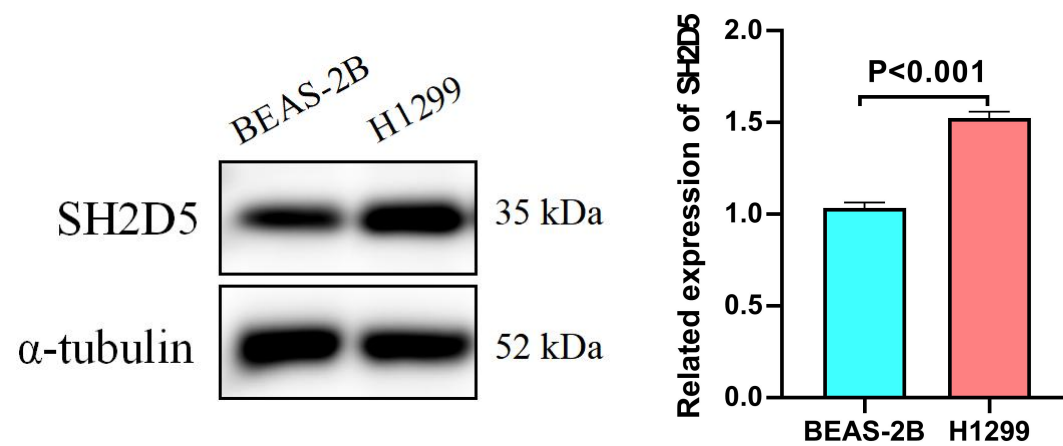**C**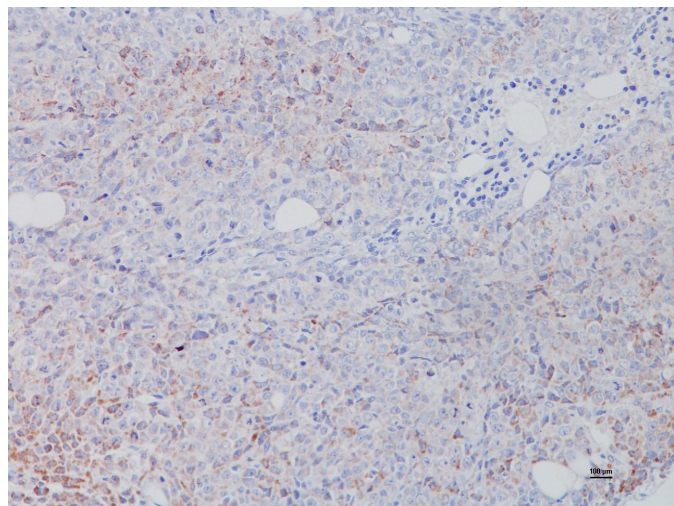

Normal

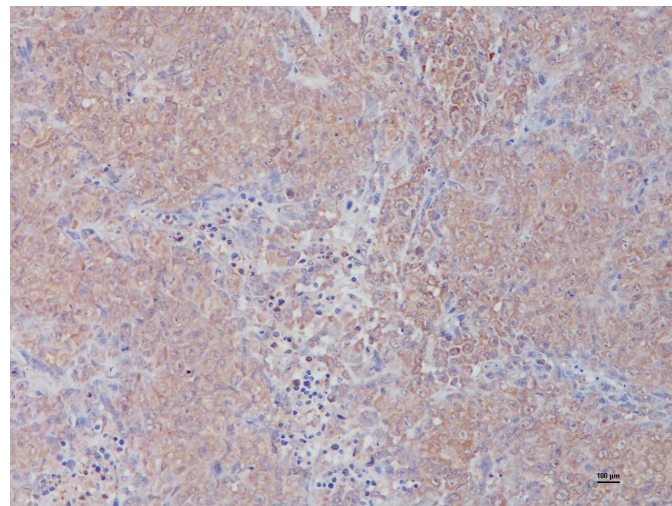

Tumor

**D**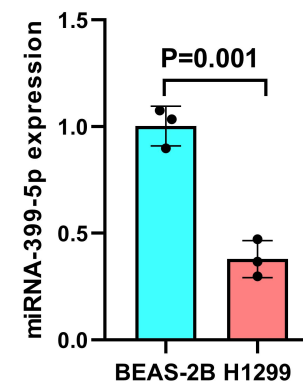

Supplement: Figure S2 — (A) Relative SH2D5 expression levels in BEAS-2B and H1299 cell lines. (B) Western blot analysis of the protein expression of SH2D5 overexpression in BEAS-2B and H1299 cell lines. The left side shows the Western blotting result and the right side shows the statistical diagram. (C) Relative miRNA-339-5p expression levels in BEAS-2B and H1299 cell lines. [file peerj-11-15238-s004.pdf]

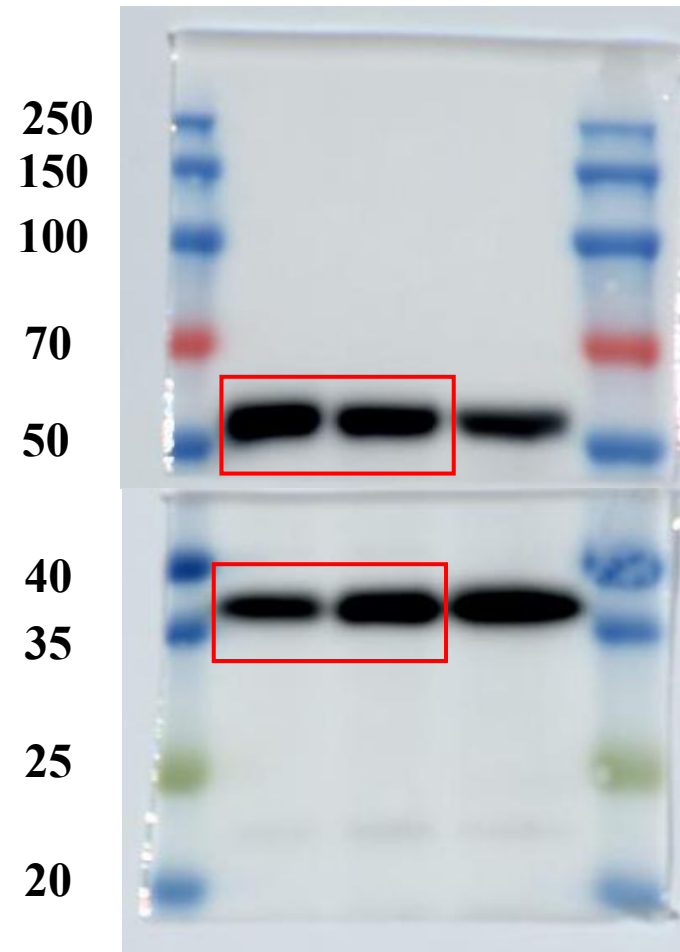

$\alpha$ -tubulin 52 kDa

SH2D5 35 kDa

Supplement: Supplemental Information 7 [file peerj-11-15238-s007.pdf]
